# Fermented wheat germ extract - nutritional supplement or anticancer drug?

**DOI:** 10.1186/1475-2891-10-89

**Published:** 2011-09-05

**Authors:** Thomas Mueller, Wieland Voigt

**Affiliations:** 1 University of Halle, Department of Internal Medicine, Oncology/Hematology and Hemostaseology, Ernst-Grube Str. 40, 06120 Halle/Saale, Germany

**Keywords:** Fermented wheat germ extract, *in vitro *effects, *in vivo *effects, clinical activity

## Abstract

**Background:**

Fermented wheat germ extract (FWGE) is a multisubstance composition and, besides others, contains 2-methoxy benzoquinone and 2, 6-dimethoxy benzoquinone which are likely to exert some of its biological effects. FWGE interferes with anaerobic glycolysis, pentose cycle and ribonucleotide reductase. It has significant antiproliferative effects and kills tumor cells by the induction of apoptosis via the caspase-poly [ADP-ribose] polymerase-pathway. FWGE interacts synergistically with a variety of different anticancer drugs and exerted antimetastatic properties in mouse models. In addition, FWGE modulates immune response by downregulation of MHC-I complex and the induction of TNF-α and various interleukins. Data in the F-344 rat model provide evidence for a colon cancer preventing effect of FWGE.

Clinical data from a randomized phase II trial in melanoma patients indicate a significant benefit for patients treated with dacarbazine in combination with FWGE in terms of progression free survival (PFS) and overall survival (OS). Similarly, data from studies in colorectal cancer suggested a benefit of FWGE treatment. Besides extension of OS and PFS, FWGE improved the quality of life in several studies.

**Conclusion:**

In conclusion, available data so far, justify the use of FWGE as a non-prescription medical nutriment for cancer patients. Further randomized, controlled and large scale clinical studies are mandatory, to further clarify the value of FWGE as a drug component of future chemotherapy regimens.

## Background

The majority of cancer patients eventually suffers from tumor cachexia and anorexia during their course of disease [1]. The pathogenesis of tumor cachexia is still not fully understood but a multifactorial process including pro-inflammatory cytokines like IL-6 or TNF-α, neuroendocrine hormones, downregulation of IGF-1 and tumor proteolysis inducing factor is assumed [1,2]. Clinically, the process of tumor cachexia leads to emaciation, weakness and fatigue with significant impact on quality of life [1]. On the cellular level, beside others tumor cachexia results in a suppression of the immunological response and metabolic dysfunction [3]. In addition, recent data have identified tumor cachexia as an independent predictor of shorter survival and suggest that cancer patients with significant body weight loss have an increased risk of treatment failure [2,3]. These findings support the early therapeutic intervention like the use of specific nutriments even before the onset of significant body weight loss, particularly in cancers well known to induce severe cachexia like pancreatic cancer [3]. One of the nutrition supplements for cancer patients in current clinical use is fermented wheat germ extract (FWGE) which is available as an over the counter dietary supplement in several parts of the world under the brand name Avemar^®^. Like typical for several other nutrition supplements fermented wheat germ extract contains hundreds to thousands of different molecules but based on recent studies with various extracts from fermented wheat germ it is currently assumed that the two quinones 2-methoxy benzoquinone and 2, 6-dimethoxy benzoquinone which are present in wheat germ as glucosides were likely to be responsible for some of the biological properties of FWGE [3,4]. The patented production process of FWGE consists of the extraction of wheat germs, fermentation by Saccharomyces cerevisiae, separation of the fermentation liquid, drying and granulation. This well defined and fingerprint chromatography controlled production process results in a laboratory-standardized compound [3]. The availability of large quantities of fermented wheat germ extract allowed an intensified preclinical and clinical research resulting in the characterization of several mechanisms of action and it provided evidence for potential versatile clinical activity. Despite its manifold activities, no meaningful toxicity, mutagenicity or genotoxicity has been observed [5]. In addition to its single agent effects, fermented wheat germ extract appeared not to increase toxicity or to reduce activity of conventional chemotherapy [6,7]

The objective of this review is to summarize and discuss the data available on the modes of action and preclinical and clinical activity of Avemar^® ^in malignant disease.

### Mechanisms of action

#### Metabolic effect

In comparison to normal tissue, cancer cells display a hypermetabolic state with in particular upregulated utilization of glucose and production of large amounts of lactate [8]. Glucose serves as substrate for the non-oxidative pathway of ribose synthesis which is a prerequisite for the increased nucleic acid production in the rapidly dividing tumor cells. FWGE inhibits glucose uptake in cancer cells and interferes with enzymes of the anaerobic glycolysis and PPP such as transketolase, glucose-6-phosphate dehydrogenase, lactate dehydrogenase and hexokinase which are necessary for the allocation of precursors for RNA and DNA-synthesis [9-11]. Besides the inhibition of glycolysis and PPP, FWGE impairs the allocation of precursors for DNA-synthesis by interference with ribonucleotide reductase. This protein is the key enzyme for the *de novo *synthesis of DNA and it converts ribonucleotides to deoxyribonucleotide-triphosphates, which are precursors of DNA-synthesis [12]. The inhibition of these key pathways of sugar metabolism and DNA-synthesis contributes to the proliferation inhibiting capacity of FWGE. Ribonucleotide reductase is frequently found to be upregulated in human cancer cells making it an attractive target for anticancer therapy. Like many other anticancer drugs, e.g. gemcitabine, fludarabine or clofarabine, FWGE has been demonstrated to significantly inhibit ribonucleotide reductase in HT29 human colon cancer and HL-60 human promyelocytic leukemia cell lines [13,14].

Beside its DNA-synthesis interfering effects FWGE inhibits the activity of cyclooxygenases 1 and 2 with similar potency. Cyclooxygenases are key enzymes in the synthesis of prostaglandins from arachidonic acid. Prostaglandins regulate inflammatory processes and this might explain the activity of FWGE in rheumatoid arthritis [15].

#### Antiproliferative effect

Several research groups, including ours, have studied the antiproliferative activity of FWGE in human tumor models *in vitro *and *in vivo *[8,10,13,16-22]. In a large *in vitro *anticancer drug screen we found FWGE to possess potential antitumor activity in colon, testis, thyroid, ovary, NSCLC, breast, gastric, head and neck, hepatoma, glioblastoma, melanoma, cervix and neuroblastoma human cancer cell lines. In this set of data, the observed IC50 ranged from 0.04 to 0.7 mg/ml which is in accordance with reports from other groups [6,23]. In different cell line or xenograft models, FWGE was found to reduce tumor growth in a dose dependent manner [9,10,13,18-20,23,24]. It appears that FWGE kills cells by the induction of apoptosis via the caspase - PARP-pathway [9,17].

Of note, beside its single agent activity, FWGE augmented the activity of tamoxifen in estrogen receptor-positive breast cancer cells [23,25,26]. In in vitro studies from our group in colon cancer cell lines, combinations of 5-FU, oxaliplatin and irinotecan with FWGE exerted additive to synergistic drug interactions [6]. In *in vivo *studies, combinations of FWGE and 5-FU or DTIC resulted in synergistic drug interaction in colon (human HCR-25 cell line) and melanoma (murine B16 cell line) mouse tumor models with reduction of tumor size in the colon model and reduction of incidence of metastasis formation in both models [19,26]. Szende et al. evaluated the combined treatment of FWGE with dacarbazine, adriablastin, or 5-FU in MCF-7, HepG2 or Vero cell line, as well as vinorelbine, cyclophosphamide and doxorubicin in 3LL-HH tumor bearing mice, and found no significant drug interaction, in particular no antagonism of the cytostatic drug effect by FWGE [7]. In addition, combination of FWGE and chemotherapy did not increase toxicity in mice [7].

#### Antimetastatic effect

An antimetastatic effect of FWGE alone or in combination with cytostatic drugs was described by Hidvegi et al. in a spleen-liver or muscle-lung mouse metastasis model using 3LL-HH, B16 and HCR-25 cell lines [19]. In all three mouse models, the treatment with FWGE orally at 3 g/kg daily dosage resulted in a significant reduction of liver or lung metastasis as compared to control mice [19]. Furthermore, combined treatment of FWGE and either DTIC or 5-FU synergistically decreased the number of metastasis in the melanoma (B16) and colon cancer (C38) model [19]. In another study reported earlier by Hidvegi et al. FWGE alone or in combination with vitamin C reduced the incidence of metastasis in 3LL-HH (variant of Lewis lung carcinoma), B16 (murine melanoma), RWT-M (rat nephroblastoma) and HCR25 (human colon cancer) in mouse models [20]. It's worthy to note that combined treatment with FWGE and vitamin C exerted an inferior effect compared to the FWGE alone group in the B16 melanoma model [20].

#### Immunological effect

Several of the observed preclinical and clinical effects of FWGE cannot be attributed to its direct antiproliferative or metabolic effect, but rather to a modulation of immune response.

One common way for tumor cells to evade destruction by the immune system - particularly natural killer cell activity - is the overexpression of the MHC-I complex. It could be shown, that FWGE significantly downregulated the cell surface MHC class I proteins [21]. This process was rather selective for malignant T- and B-cells since healthy peripheral blood mononuclear cells remained unaffected. The downregulation of the MHC class I protein was preceded by specific tyrosine phosphorylation of several intracellular proteins and increase of intracellular calcium concentrations [21].

Tumors follow multiple strategies to escape the immunological response. Like overexpression of MHC class I protein, the decreased expression of ICAM-1 protein on the endothelial cells of solid tumor vessels impairs access of the immune system to the tumor [27]. Reduced ICAM-1 expression impairs leukocyte migration through the vessel membrane and thereby inhibits tumor leukocyte infiltration. FWGE was shown to upregulate the expression of ICAM-1 on tumor endothelial cells and to synergize these effects of tumor necrosis factor alpha [16]. In the same study, FWGE induced the production of cytokines. Up to the toxic threshold, FWGE induced the secretion of TNF-α dose-dependently in myeloid but not in lymphoid cells [16]. Furthermore, Telekes et al. reported an induction of interleukins such as IL-1α, IL-2, IL-5 and IL-6 [16].

Further hints for an immune stimulatory effect of FWGE came from skin graft experiments reported by Hidvegi et al. [19,28]. They used C57B1/10 mice as recipient and the B10 LP sub strain as donor of skin. The recipient group was subdivided into a group of thymectomized and non-thymectomized mice. After skin transplantation, mice were either treated with FWGE, or not, and time to skin rejection was measured. As the main finding, FWGE significantly shortened the time of skin rejection in the thymectomized mice, indicating an ability of FWGE to decrease the immune deficiency caused by thymectomy [19,28].

#### Prevention of colon cancer

The effect of FWGE on colon carcinogenesis induced by azoxymethane was studied in F-344 rats. Treatment of rats with azoxymethane alone induced colon tumors in 83% of all rats. However, if rats were treated with a combination of azoxymethane and FWGE, the incidence of colon tumors shrunk to 44.8%. This effect was paralleled by a reduction in the number of aberrant crypt foci in approximately the same magnitude [24]. Despite these significant results the exact molecular mechanism of this observation is yet to be established.

### Clinical trials

Preclinical data suggested potential activity of FWGE in colon cancer and melanoma [19]. Consequently, clinical research was focused on these two entities. A randomized open label phase II study was performed in stage III melanoma patients comparing adjuvant treatment with DTIC with a combination of DTIC plus FWGE 8.5 g p.o. once daily for a period of 12 month [29]. 52 patients were treated and followed up, for up to seven years. There was no statistical difference in the baseline parameters of the two groups. The primary study end point was progression-free survival. Overall, results were encouraging, with an observed PFS for the combination group of 55.8 months vs. 29.9 for DTIC alone, and OS of 66.2 months for the combination group and 44.7 months for DTIC alone. These results reached statistical significance with a p-value < 0.05. In addition to the improved survival data, the incidence of adverse events related to anticancer treatment was lower in the combination arm [29].

In 1998 two pilot scale phase II studies and one large scale phase III study were launched in colorectal cancer [30-32]. All study results were in favor of the combination group including FWGE treatment. The study performed by Jakab et al. enrolled 170 patients in a phase III design. The study was multicentric, and had an open label and cohort design. Allocation to each treatment arm was made by patient's choice. Standard treatment consisted of radical surgery plus radiotherapy and/or chemotherapy (Mayo Clinic regimen). Primary end point was progression-free survival. Due to the allocation design, the two treatment groups were not balanced, with more patients of advanced tumor stage (including stage IV) in the FWGE group [32]. PFS and OS were more favourable for the FWGE group [32]. In a multivariate analysis, tumor stage and treatment with FWGE were the only significant predictors of survival [32]. The side effects reported by Jakab et al. were overall mild and consisted of diarrhea, nausea and vomiting, flatulence, repletion, soft stool and constipation in less than 10% of all patients [32].

Beside the issue of potential prolongation of PFS and OS by the addition of FWGE to chemotherapy regimens, its influence on the quality of life of cancer patients was studied amongst others in lung and breast cancer [15].

In lung cancer, a study was performed with enrollment of 16 patients receiving either chemotherapy and/or radiotherapy. FWGE was administered for 8 months and quality of life was assessed using the EORTC QLQ-C30 questionnaire. After 12 weeks of administration of FWGE, an improvement of quality of life was reported in global state of health and fatigue. This improvement maintained throughout the whole observation period [15]. A multicentric quality of life study was launched in breast cancer. 55 patients were enrolled and changes of quality of life were detected using the EORTC QLQ-C30 questionnaire. The mean observation period was 32 months. Several components of quality of life showed significant improvement while on supportive therapy with FWGE. 3 months after start of treatment improvement reached significance (p < 0.05) in physical functions, emotional functions, global state of health, nausea and vomiting, insomnia and constipation. These improvements remained stable throughout the entire period of treatment [15].

In a phase III open-label, multicentric study, 60 head and neck cancer patients (stage III-IV) were divided into two groups, one treated with standard anti-cancer therapy (SAT) alone, while the other group was treated with SAT plus FWGE [33]. After 2 months, the Spitzer's Quality of Life Index (SQOLI) was significantly improved, and the levels of the circulating hydroperoxides were significantly reduced in the FWGE group [33].

In a preclinical study, FWGE accelerated the regeneration of thrombocytes and reticulocytes in sublethally irradiated or cyclophosphamide treated mice [34]. Thus, an open-label, matched-pair, pilot clinical trial was carried out to test whether the co-administration of FWGE with cytotoxic drugs, and the continued administration of FWGE on its own, can reduce the incidence of treatment-related febrile neutropenia in children with solid cancers, compared with the same treatments without FWGE [35]. During the treatment and the follow-up period, there was no progression of the malignant disease, whereas at end-point, the number and frequency of febrile neutropenic events significantly differed between the two groups: 30 febrile neutropenic episodes (24.8%) in the FWGE plus cytotoxic drugs group versus 46 (43.4%) in the cytotoxic drugs group (Wilcoxon signed rank test, p < 0.05). The authors concluded that FWGE may therefore be recommended to reduce the incidence of treatment-related febrile neutropenia in children with solid cancers [35].

## Discussion

Nutritional supplements are being used by a significant amount of cancer patients to augment their conventional cancer therapy or to improve cancer or therapy-related symptoms. Some of these nutraceuticals are approved as dietary food for special medical purposes for cancer patients. To this group of nutrition supplements belongs the FWGE (Avemar^®^).

Preclinical *in vitro *and *in vivo *data suggested antiproliferative, antimetastatic and immunological effects of FWGE [6,13,15,19,21]. In addition, the interference of FWGE with several metabolic pathways has been well described [9,10,13,15]. The battery of preclinical data prompted the performance of some clinical studies, such as in melanoma and colorectal cancer. Taken together, these studies suggested that FWGE has the potential to improve response to chemotherapy and thereby to extend the PFS and OS of cancer patients even in advanced stages [29-32]. These interesting set of preclinical and clinical data raise the question of the status of FWGE: nutritional supplement or anticancer drug?

Looking at the summarized data on the preclinical and clinical activity profile of FWGE, it seems to be a promising candidate for further clinical evaluation in the treatment of cancer patients. The interference with the hypermetabolic state of cancer cells resulting in inhibition of the necessary pathways to produce the precursors of DNA-synthesis makes the substance somewhat selective for malignant cells. This notion is supported by data from Comin-Anduix et al. who reported an about 50 fold higher IC50 for FWGE in peripheral blood lymphocytes as compared to Jurkat cells [9]. This relatively broad therapeutic window is also corroborated by the lack of significant side effects of FWGE in the *in vivo *studies and in clinical trials [19,29,32]. From the clinical and preclinical data, it is suggested that FWGE has single agent activity and appears to modulate (synergize) the effect of commonly used cytostatic and other anticancer drugs [6,18,19,21,23,29,32]. The inhibition or lack of G6PDH sensitizes cells to radiation-induced apoptosis [36,37]. Thus, a combination of FWGE, a potent inhibitor of several enzymes of the PPP including G6PDH, with radiotherapy appears to be worth testing. An additional target of FWGE involved in DNA-synthesis is ribonucleotide reductase, which is upregulated in several cancers and therefore is considered an important target in cancer chemotherapy [14]. Clinically active drugs like fludarabine or cytarabine and gemcitabine exert at least, in part, their cytotoxic activity by inhibiting ribonucleotide reductase [14].

FWGE is a multisubstance composition as reflected by its specific HPLC fingerprint spectra and the exact chemical composition is yet unknown. Two major components are the quinones 2-methoxy benzoquinone and 2, 6-dimethoxy benzoquinone. These two components are considered to be mainly involved in the antiproliferative and metabolic activity of FWGE. However, it's suggested by data from Hidvegi et al. using a skin graft model that components other than the two benzoquinones are responsible for the immune stimulatory activity of FWGE [28]. On the other hand, Fajka-Boja et al. demonstrated that FWGE downregulated MHC-I in malignant B and T cell lines by a mechanism which is yet not clear in detail. Likely, the quinone fraction of FWGE is at least, in part, involved in this phenomenon since comparative studies in Jurkat cells lead to a 70% decrease of MHC-I when treated with quinones alone, and 90% when treated with the complete extract [21]. Based on these observations, it's tempting to speculate that the skin graft rejection observed by Hidvegi et al. could be attributed to other known immune modulating effects of FWGE like induction of TNF-α, IL-2 and IL-4 or INF-γ [16,38]. In addition, downregulation of MHC-I protein expression was accompanied by increase of intracellular calcium concentration and tyrosine phosphorylation of some yet unidentified proteins [21]. Overall, it appears that FWGE exerts its cytotoxic and antimetastatic effects by a combination of direct cytotoxicity and immune modulation.

In clinical use, FWGE is very well tolerated at the recommended dose and posseses a broad therapeutic window [26]. Besides its antitumoral activity, FWGE seems to exert additional effects leading to improvement of quality of life [15]. This might be in part explained by the metabolic changes in cancer patients induced by FWGE. Its interference with the PPP switching from nonoxidative to oxidative reaction leads to decreased nucleic acid synthesis from glucose to direct glucose oxidation and lipid synthesis. This in turn may result in weight gain of the patient and improvement of body shape [15,39]. It is interesting to note, that FWGE has a similar metabolic effect and targets components of the PPP like the well known targeted drug imatinib (Glivec^®^) [40].

The clinical properties of FWGE in cancer patients were observed in a limited number of small and large-scale studies in different kind of cancers. However, except the study conducted by Demidov et al., all other studies were not randomized and do not meet the standards of clinical trials for drug registration. Thus, the potential anticancer drug effect of FWGE cannot be considered as clinically proven. Further well-conducted, randomized and large-scale trials are mandatory to prove the supposed effects of FWGE.

In conclusion, available data suggest that FWGE has an interesting preclinical and clinical activity profile with no toxicity. It appears to exert its effects by a battery of diverse mechanisms, likely because of its multi-substance composition. The use of FWGE as a non-prescription medical nutriment for cancer patients seems maintainable and combined use with chemotherapy appears feasible. However, current data do not justify the use of FWGE as an anticancer drug. Further randomized, well controlled and large scale clinical studies meeting the demands of clinical trials for drug registration are mandatory and warranted to further clarify the potential value of FWGE as a drug component of future chemotherapy regimens.

## Abbreviations

DTIC: Dacarbazine; DNA: Deoxyribonucleic acid; EORTC: European Organisation for Research and Treatment of Cancer; FDA: Food and Drug Administration; 5-FU: 5-Fluorouracil; FWGE: Fermented wheat germ extract; HPLC: High Pressure Liquid Chromatography; ICAM-1: intercellular adhesion molecule 1; IGF-1: insulin-like growth factor 1; MHC: Major histocompatibility complex; NSCLC: Non small cell lung cancer; OS: Overall survival; PARP: Poly (ADP-ribose) polymerase; PFS: Progression-free survival; PPP: Pentose phosphate pathway; TNF-α: tumor necrosis factor α.

## Competing interests

The authors declare that they have no competing interests.

## Authors' contributions

TM and WV equally contributed to the preparation of the manuscript.

Both authors read and approved the final manuscript.

## Authors' information

Thomas Mueller, PhD is a biochemist and head of tumorbiology at the department of internal medicine, oncology/hematology and hemostaseology

Wieland Voigt, MD is a medical oncologist and associate professor. He serves as a senior physician and head of experimental oncology at the department of internal medicine, oncology/hematology and hemostaseology
